# Genetic exploration of roles of acid‐sensing ion channel subtypes in neurosensory mechanotransduction including proprioception

**DOI:** 10.1113/EP090762

**Published:** 2023-07-25

**Authors:** Yi‐Chen Lin, Cheng‐Han Lee, Jia‐Ying Sung, Chih‐Cheng Chen

**Affiliations:** ^1^ Department of Neurology, Wan Fang Hospital Taipei Medical University Taipei Taiwan; ^2^ The Ph.D. Program for Translational Medicine Taipei Medical University and Academia Sinica New Taipei City Taiwan; ^3^ Taipei Neuroscience Institute Taipei Medical University New Taipei City Taiwan; ^4^ Institute of Biomedical Sciences Academia Sinica Taipei Taiwan; ^5^ Neuroscience Program of Academia Sinica Academia Sinica Taipei Taiwan; ^6^ Department of Neurology, School of Medicine, College of Medicine Taipei Medical University Taipei Taiwan; ^7^ Taiwan Mouse Clinic – National Comprehensive Mouse Phenotyping and Drug Testing Center Academia Sinica Taipei Taiwan; ^8^ TMU Neuroscience Research Center, Taipei Medical University New Taipei City Taiwan

**Keywords:** ASIC, ASIC1a, ASIC3, proprioception, ultrasound

## Abstract

Although acid‐sensing ion channels (ASICs) are proton‐gated ion channels responsible for sensing tissue acidosis, accumulating evidence has shown that ASICs are also involved in neurosensory mechanotransduction. However, in contrast to Piezo ion channels, evidence of ASICs as mechanically gated ion channels has not been found using conventional mechanoclamp approaches. Instead, ASICs are involved in the tether model of mechanotransduction, with the channels gated via tethering elements of extracellular matrix and intracellular cytoskeletons. Methods using substrate deformation‐driven neurite stretch and micropipette‐guided ultrasound were developed to reveal the roles of ASIC3 and ASIC1a, respectively. Here we summarize the evidence supporting the roles of ASICs in neurosensory mechanotransduction in knockout mouse models of ASIC subtypes and provide insight to further probe their roles in proprioception.

## Abstract (highlights)

- **What is the topic of this review?**
  The roles of acid‐sensing ion channels in mechanotransduction, including proprioception, along with new investigative approaches.
- **What advances does it highlight?**
  New methods such as substrate deformation‐driven neurite stretch and micropipette‐guided ultrasound can help in studying the functions and electrophysiological properties of ASICs essential for a tether model of mechanotransduction, especially conduction of proprioceptive signals from the muscle spindle to specific groups of dorsal root ganglion neurons.

## 1 INTRODUCTION

Acid‐sensing ion channels (ASICs) are proton‐gated, mainly sodium‐ but also calcium‐permeable channels that belong to the degenerin/epithelial sodium channel family (Deg/ENaC). Since ASIC2 was first cloned by Price et al. (1996), at least six ASIC subtypes have been discovered, including ASIC1a, ASIC1b, ASIC2a, ASIC2b, ASIC3 and ASIC4 (Lin et al., 2015). ASICs can be activated by protons, non‐proton ligands (e.g., arachidonic acid, lysophosphatidylcholine, carbon dioxide, lactate) and mechanical stimulation (Cheng et al., 2018). They are involved in different sensory functions, including nociception, sour taste, interoceptions, pruriception (itch sensation) (Peng et al., 2015) and sngception (a somatosensory function of acid‐sensing) (Hung et al., 2023; Lin et al., 2018). Also, evidence has shown that ASICs are involved in neurosensory mechanotransduction (Chen & Wong, 2013; Cheng et al., 2018). Except for ASIC4, ASICs are expressed in mechanoreceptors of dorsal root ganglia (DRG) (Lin et al., 2016). In this article, we focus on ASIC‐related mechanotransduction, including proprioception.

## 2 ROLES OF ASICS IN NEUROSENSORY MECHANOTRANSDUCTION

**FIGURE 1:**
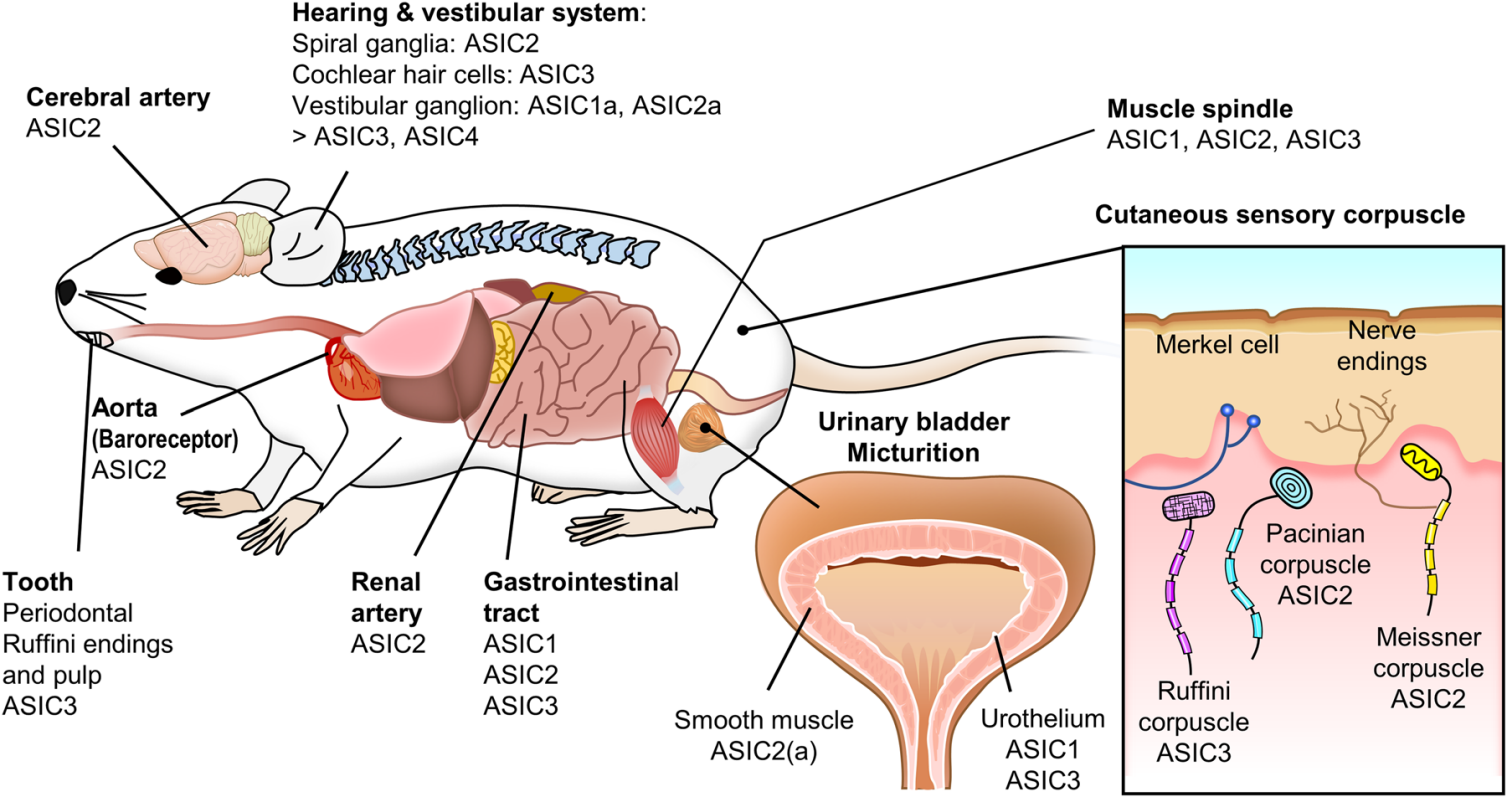
Roles of ASICs in neurosensory mechanotransduction. The distribution and expression of ASICs associated with mechanotransduction, including proprioception, in mice. ASIC1, ASIC2 and ASIC3 are involved in mechanosensory conduction and widely distributed in the aorta, cerebral and renal arteries, ears, teeth, cutaneous regions, muscles, gastrointestinal tract and urinary bladder.

**FIGURE 2:**
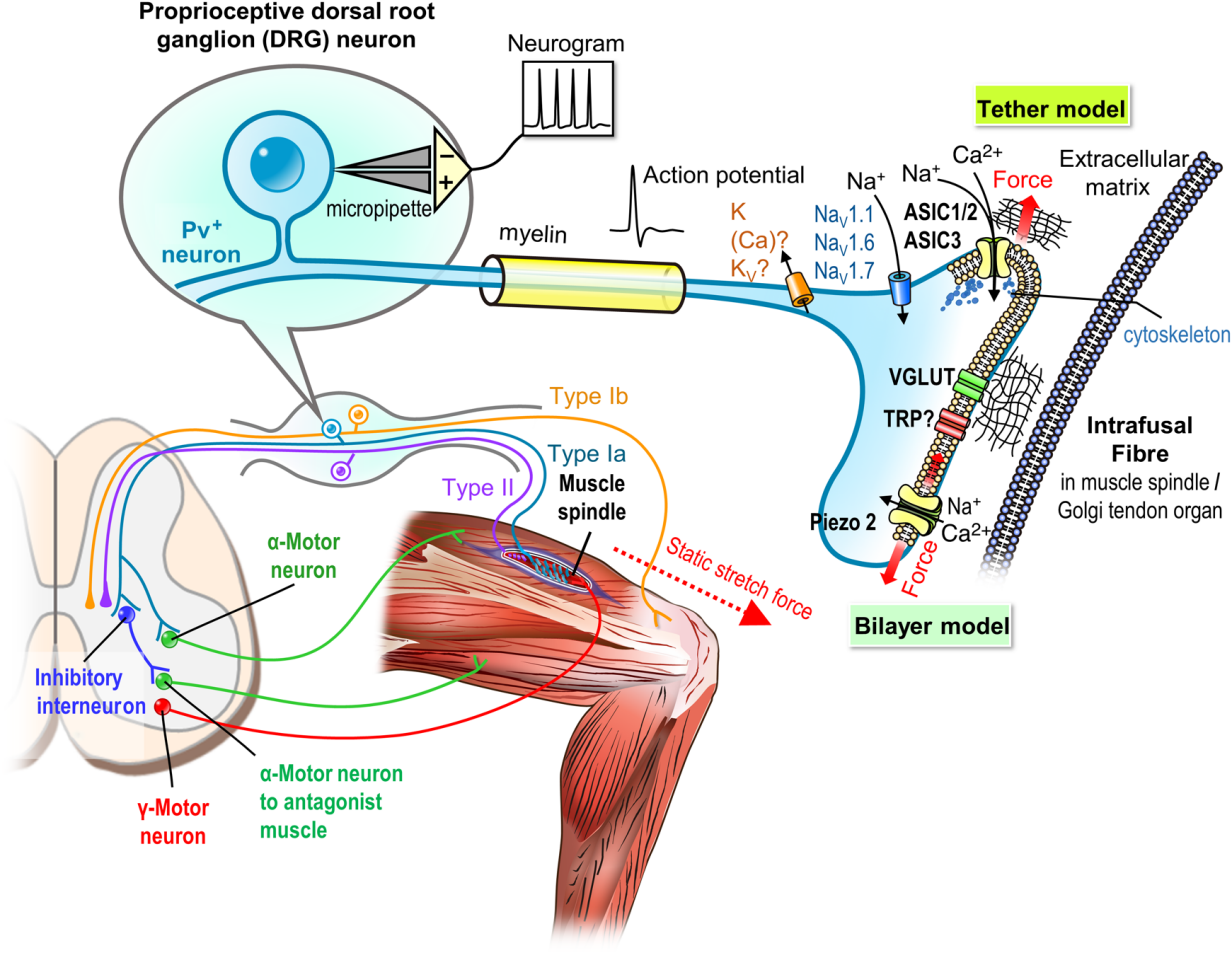
The molecular mechanism of proprioception in muscle spindle and afferent nerve terminals with trajectories to dorsal root ganglia (DRG), spinal cord and motor neuron synapse. Parvalbumin‐expressing (Pv^+^) neurons are the major subgroup in DRG for proprioceptive information from the muscle spindles or Golgi tendon organs. The upper panel illustrates that ASICs and Piezo2 are key mechanosensory channels for different mechanical stimulation (shown in tether model and bilayer model, respectively; the red arrows indicate the direction of mechanical force applied to the extracellular matrix and membrane). The role of TRP remains unclear. VGLUT, Na_v_ 1.1/1.6/1.7 and potassium channels participate in the propagation of the action potential. The lower panel reveals the representative stretch reflex loop. When muscles are passively stretched (shown by red dashed arrow), proprioceptive signals from type Ia fibre in the muscle spindle are conducted to the DRG neuron, the dorsal horn of the spinal cord, and form the synapse with inhibitory interneurons or directly to the α‐motor neuron, which innervates extrafusal muscle fibres. The interneurons release inhibitory neurotransmitters at the synapse with another α‐motor neuron, innervating antagonist muscle fibres. (Completed loops of type Ib fibres from the Golgi tendon organ and type II fibres from intrafusal chains are not shown here.).

ASICs are widely expressed in the peripheral nervous system, such as sensory neurons of the trigeminal ganglion (Ichikawa & Sugimoto, 2002), mesencephalic trigeminal nucleus (Nakamura & Jang, 2014), spiral ganglion (Peng et al., 2004), nodose ganglion (Lu et al., 2009) and DRG (Papalampropoulou‐Tsiridou et al., 2022; Wu et al., 2021). These sensory afferents innervate tissues all over the body, including the head, eyes (Meng et al., 2015; Stankowska et al., 2018), teeth (Rahman et al., 2011), muscle (Gautam & Benson, 2013; Simon et al., 2010), skin (Cobo et al., 2020; García‐Añoveros et al., 2001; Price et al., 2000) and visceral organs (Dusenkova et al., 2014; Kobayashi et al., 2009; Montalbetti et al., 2018; Page et al., 2005), to monitor mechanical stimuli (Figures 1 and 2). To understand the roles of ASICs in neurosensory mechanotransduction, genetic approaches have been widely used to probe the physiological phenotypes in different ASIC subtype‐specific knockout mice (Table 1).

**TABLE 1:** Effect of ASICs knockout involving mechanotransduction, including proprioception.

Physiological function	ASIC1a	ASIC2	ASIC3
Cutaneous sensory mechanoreceptors			
RA mechanoreceptor sensitivity	↔ (Page et al., 2004)	↓ (Price et al., 2000), ↔ (Roza et al., 2004)	↑ (Price et al., 2001)
SA mechanoreceptor sensitivity	↔ (Page et al., 2004)	↓ (Price et al., 2000)	↔ (Price et al., 2001)
A‐fibre mechanonociceptor activity	↔ (Page et al., 2004)	↔ (Price et al., 2000)	↓ (Price et al., 2001)
D‐hair afferents	↔ (Page et al., 2004)	↔ (Price et al., 2000)	↔ (Price et al., 2001)
C‐fibre afferents	↔ (Page et al., 2004)	↔ (Price et al., 2000)	↔ (Price et al., 2001)
Baroreceptor			
Baroreflex reflex and afferent to nodose ganglion		↓ (Gannon et al., 2008; Lu et al., 2009)	
Cerebral artery pressure‐induced myogenic constriction against hypertension		↓ (Gannon et al., 2008)	
Renal artery myogenic vasoconstriction and regulation of hypertension		↓ (Gannon et al., 2015; Lu et al., 2022)	
Mechanoreceptors of gastrointestinal tract			
Gastroesophageal tension afferent	↑ (Page et al., 2004, 2005)	↓ (Page et al., 2005)	↓ (Bielefeldt & Davis, 2008; Page et al., 2005)
Gastroesophageal mucosal afferent	↑ (Page et al., 2004, 2005)	↑ (Page et al., 2005)	↔ (Page et al., 2005)
Colon serosal afferent	↑ (Page et al., 2004, 2005)	↑ (Page et al., 2005)	↓ (Page et al., 2005)
Colon mesenteric afferent	↑ (Page et al., 2004, 2005)	↔ (Page et al., 2005)	↓ (Page et al., 2005)
Colon muscular‐mucosal afferents			↓ (Jones et al., 2005)
Colon RA mechanosensory afferents and related nociception		↔ (Roza et al., 2004)	
Mechanoreceptors of urinary bladder			
Bladder overactivity/hyperreflexia			↓ (Montalbetti et al., 2018)
Cardiovascular system			
Afferent activity to NG from atrium‐induced blood volume expansion and diuresis			↓ (Lee et al., 2011)
Cardiac autonomic responsiveness			↓ (Cheng et al., 2014)
Mechanoreceptors of auditory system			
Age‐dependent hearing loss (hair cells in cochlear)			↓ (Hildebrand et al., 2004; Wu et al., 2009)
Noise‐induced temporary threshold shifts (spiral ganglia)		↓ (Peng et al., 2004; Roza et al., 2004)	
Proprioceptors (substrate deformation‐driven neurite stretch)			
Stretch‐induced mechanosensitive currents			↓ (Lin et al., 2016)

*Note*: Summary of the effect on the physiological function under the common types of ASIC knockout. ASICs are critical for mechanotransduction in the cutaneous somatosensory system, blood vessel wall, gastrointestinal tract, urinary tract, cardiovascular system and ears. Some of the physiological effects studied with ASIC agonists or antagonists without data on genetic knockout are not listed in the table. Abbreviations: ↑, increase or enhancing; ↓, decrease or impairing.; ↔, no change; NG, nodose ganglion; RA, rapid adapting; RV, right ventricle, SA, slow adapting.

### 2.1 Cutaneous sensory mechanoreceptors

ASIC2 is highly expressed in cutaneous mechanoreceptors (hair follicle/vibrissal afferents, penicillate endings, intraepidermal nerve endings, Merkel cells and Meissner corpuscle) for specific sensory modalities (García‐Añoveros et al., 2001). ASIC2 knockout conferred a reduced sensitivity of rapidly adapting and slowly adapting mechanoreceptors (Price et al., 2000). Of note, some researchers suggested that cutaneous mechanosensation is not impaired in ASIC2‐knockout mice (Roza et al., 2004). Of note, ASIC3‐knockout mice showed enhanced sensitivity of rapidly adapting mechanoreceptors but reduced sensitivity of A‐fibre mechanoreceptors (Price et al., 2001). Thus, the exact role of ASIC2 and ASIC3 in cutaneous mechanoreceptors is still controversial because of discrepant results between studies or differential effects between mechanoreceptor subtypes.

### 2.2 Baroreceptors

Previous studies have shown that ASIC2, possibly combined with other channels including ENaCs, transient receptor potential (TRP), and Piezo channels, is required for transducing the myogenic response to pressure in the blood vessel wall, with involvement in the baroreflex. ASIC2 knockout mice present a compromised baroreflex response and nodose ganglion mechanosensitivity. In cerebral (Gannon et al., 2008) and renal arteries (Gannon et al., 2015), ASIC2‐knockout mice exhibited impaired pressure‐induced myogenic constriction against hypertension.

ASIC3 was discovered as a unique and essential channel expressed in the atrium to transduce the signals related to ‘blood volume change’. Atrial ASIC3 is responsive to blood volume expansion and is associated with the activation of nodose ganglia and DRG neurons to induce atrial natriuretic peptide secretion and diuresis (Lee et al., 2011). These studies suggest a role for ASICs in sensing pressure or volume changes in the cardiovascular system.

### 2.3 Mechanoreceptors of the gastrointestinal tract

The neurophysiological roles of ASICs in afferents innervating the stomach and oesophagus have been established. In comparing ASIC1a‐knockout mice with wild‐type mice, the stimulus–response curve showed significantly increased activity from both tension‐sensing and mucosal afferents (Page et al., 2004, 2005, 2004, 2022). Gastric emptying was prolonged, but the faecal output was not affected in ASIC1‐knockout mice (Page et al., 2004). Activity of tension‐sensing afferents was reduced but that of mucosal afferents was increased in ASIC2‐knockout mice. In ASIC3‐knockout mice, tension‐sensing afferents were distinctly suppressed in activity, with no change in the mucosal afferents (Bielefeldt & Davis, 2008; Page et al., 2005). Similarly, in applying gastro‐oesophageal passive mechanical distention, ASIC3‐knockout mice but not TRPV1‐null mice showed less activation of neurons in nodose ganglia of the vagus nerve (Bielefeldt & Davis, 2008). These tension‐sensing, ASIC3‐positive but TRPV1‐negative nodose neurons may belong to a unique group of ‘pure mechanosensitive neurons’ from the gastro‐oesophageal tract.

In the colon, ASIC1a‐knockout mice showed increased mechanical sensitivity in recordings of serosal and mesenteric afferents but no difference in faecal pellet output (Page et al., 2005). Serosal afferent fibre activity was observed in ASIC2‐knockout mice but with no significant difference in mesenteric afferent activity (Page et al., 2005). In a behavioural study, faecal pellet output decreased significantly in ASIC2‐knockout mice (Page et al., 2005). However, ASIC2‐knockout mice showed no change in high‐frequency dynamic discharge of rapidly adapting nociception induced by pressure stimulation in the colon (Roza et al., 2004). ASIC3‐knockout mice showed significantly reduced visceromotor response to colorectal distention (Jones et al., 2005). In this study, the reduction in visceromotor response was associated with decreased activity of muscular‐mucosal mechanosensory afferent fibres sensitive to circumferential stretch. Page et al. (2005) suggested that both serosal and mesenteric colon afferents were affected by lower activity in ASIC3‐knockout mice. In summary, the physiological control of motility in the upper and lower gastrointestinal tract is ASIC‐dependent.

### 2.4 Mechanoreceptors of urinary bladder

In the urinary bladder, ASIC3 is enriched in the urothelium, whereas ASIC1 and ASIC2 are abundant in the smooth muscle layer (Kobayashi et al., 2009). Although ASIC3 expression is low in the subepithelial layer in the urinary bladder (Kobayashi et al., 2009), ASIC3‐knockout mice showed reduced voiding volume and pressure required to trigger micturition (Montalbetti et al., 2018). These studies suggest that ASIC3 is involved in the neural control of bladder function, but the mechanosensing role of ASIC1 and ASIC2 in the bladder remains to be determined.

### 2.5 Mechanoreceptors of auditory and vestibular system

Both ASIC2 and ASIC3 are expressed in neurons of the spiral ganglia (Hildebrand et al., 2004; Peng et al., 2004), but only ASIC3 (Hildebrand et al., 2004) and ASIC1b (particularly at the stereocilia) (Ugawa et al., 2006) are found in hair cells of the cochlea. ASIC2 knockout did not confer significant hearing loss but did produce resistance to noise‐induced temporary threshold shifts (Peng et al., 2004; Roza et al., 2004). In contrast, ASIC3‐knockout mice showed age‐dependent hearing loss (Hildebrand et al., 2004; Wu et al., 2009). These studies suggest that ASICs in the cochlea and spiral ganglion neurons are involved in the modulation and mechanosensory conduction of hearing.

Immunohistochemical staining revealed higher levels of ASIC1a and ASIC2a than ASIC3 and ASIC4 in the vestibular ganglion (Mercado et al., 2006; Vega et al., 2009). Unlike cochlea, type I and II hair cells in crista ampullaris and macula utricle are not immunoreactive for all subtypes of ASICs. Whole‐cell patch clamp recordings showed proton‐gated currents characterized by rapid activation and desensitization (Mercado et al., 2006). Although the acid‐induced currents can be inhibited by the ASIC antagonist amiloride and acetylsalicylic acid, the mechanosensing role of ASICs in vestibular neurons has not yet been determined.

### 2.6 The periodontal ligament and pulp of teeth

Ruffini nerve endings have been found in the periodontal ligament of mouse incisors, and they originated from the trigeminal ganglia (Maeda et al., 1999). The Ruffini nerve endings could be the mechanoreceptors in the periodontal ligament (Byers, 1985; Maeda et al., 1999). ASIC3 immunoreactivity was found in Ruffini nerve endings (Rahman et al., 2011). Use of the double immunofluorescence method and retrograde tracing revealed that 33% of tooth‐pulp trigeminal ganglion neurons were ASIC3‐immunoreactive and co‐expressed with calcitonin gene‐related peptide or parvalbumin (Ichikawa & Sugimoto, 2002). Furthermore, the single‐cell RT‐PCR approach revealed ASIC3 transcripts in 67% of pulpal afferent and trigeminal ganglion neurons (Hermanstyne et al., 2008). Combined with the previous hydrodynamic theory (Brannstrom, 1986) suggesting that fluid movement within exposed dentin tubules activates sensory nerve endings, ASIC3 may play an important role in mechanotransduction of pulpal afferents and related dentin hypersensitivity.

## 3 MECHANOTRANSDUCTION OF THE BILAYER AND TETHER MODELS

Gating models of mechanically activated ion channels can be roughly divided into (1) a bilayer model, whereby ion channels may be directly gated by tension in the cell membrane (e.g., membrane deformation), and (2) a tether model, whereby ion channels are gated by tethered elements of the extracellular matrix and/or intracellular cytoskeletons to transmit the force (Nilius & Honoré, 2012). Scientists have used the whole‐cell patch clamp technique to assess the mechanically induced electrical signals via the ion channels to determine how the mechanically activated ion channels work in response to mechanical stimuli.

### 3.1 Bilayer model mechanotransduction

**FIGURE 3:**
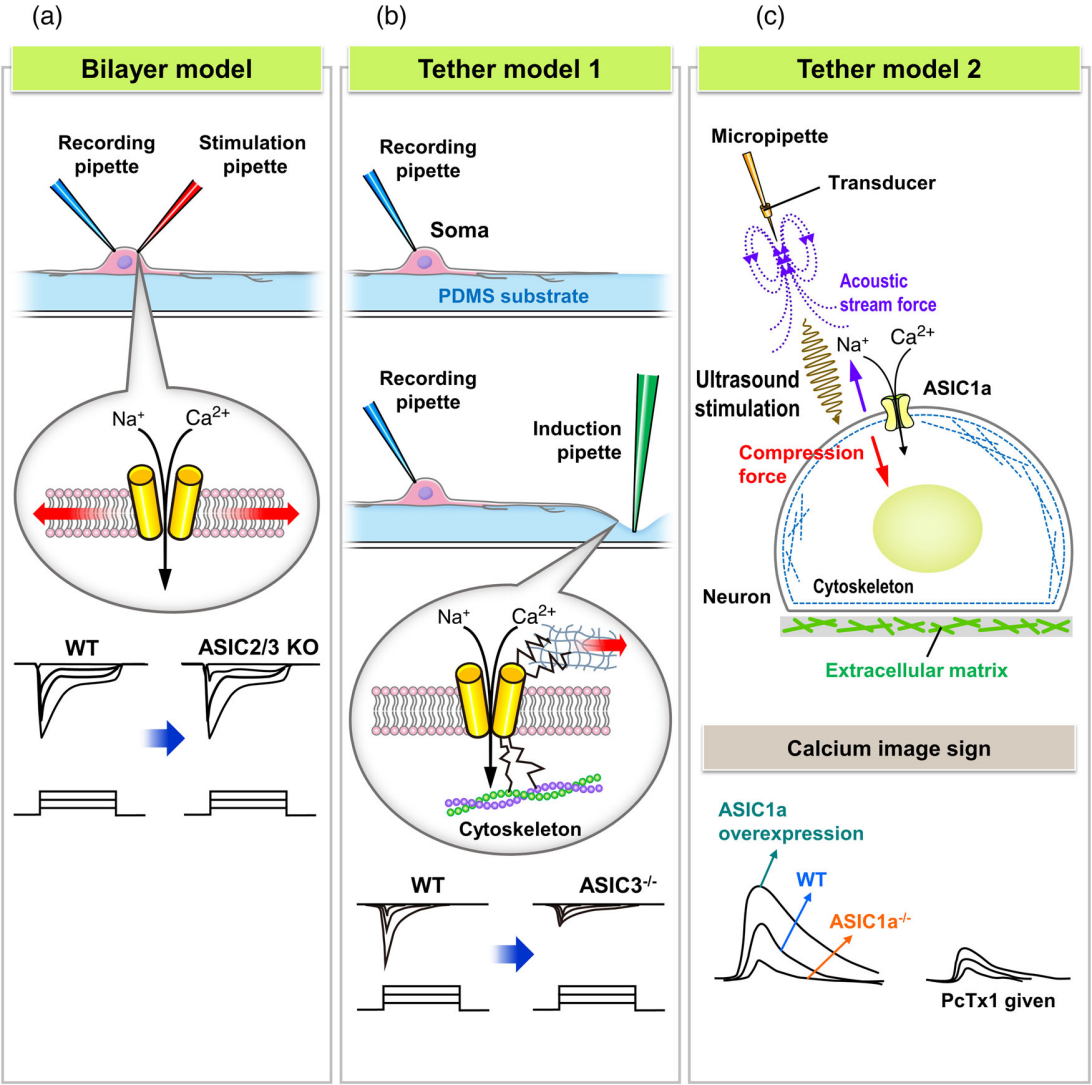
Approaches to probing mechanically activated ion channels gated via the bilayer model and tether model of mechanotransduction. (a) A schematic of the whole‐cell patch clamp recording combined with the mechanoclamp approach to probe the bilayer model of mechanotransduction. The membrane potentials can be elicited through the opening and activation of Piezo2 (not shown here) by this type of mechanical stimulation, even if the ASIC2 and ASIC3 have been knocked out. (The red arrows indicate the mechanical force.) See Drew et al. (2004). (b) A schematic representation of the whole‐cell patch clamp recording combined with substrate deformation‐mediated neurite stretch approach to probe the role of ASICs in the tether model of mechanotransduction. Mechanical force different from the bilayer model (red arrow) is applied on the extracellular matrix (shown as mesh) attaching the extracellular domain of ASIC3. ASIC3 is a crucial ion channel because a significant reduction of inward current is recorded in cultured DRG neurons with ASIC3 knockout. See Lin et al. (2016). (c) A schematic representation of the micropipette‐guided ultrasound approach to probe the tether model of mechanotransduction in calcium imaging. There are two types of mechanical force developed by micropipette‐guided ultrasound: (1) an acoustic stream near the tip of the micropipette produces a force on the extracellular matrix, ‘pulling’ the membrane outward (purple arrow) and (2) the ultrasound wave generates a compressing force toward the cell membrane. The calcium image signal intensity decreases in ASIC1a‐knockout cells but increases with ASIC1a overexpression. A similar effect of reduced signal intensity can be recorded in the presence of PcTx1, a specific ASIC1a antagonist. See Lim et al. (2021). KO, knockout; PDMS, polydimethylsiloxane; WT, wild type.

In whole‐cell patch clamp recordings, how to gently and precisely apply focal force on the neuron membrane is a challenge. For the bilayer model, suction or indentation on the plasma membrane are two common ways to apply low to high force to a cell. Therefore, to study the mechanically activated ion channels of the bilayer model, the mechanoclamp technique was developed. In this model, membrane deformation can be achieved on a micrometre scale by using an indentation pipette controlled by a piezoelectric mechanostimulator (Hao & Delmas, 2010). In this bilayer model of mechanotransduction, whole‐cell patch clamp recordings of DRG neurons revealed three major types of mechanically activated currents: rapidly adapting, intermediately adapting and slowly adapting currents. The membrane deformation‐induced mechanically activated currents in neurons or other mechanosensitive cells can be significantly reduced in knockouts of Piezo1 or 2 ion channels (Woo et al., 2014). In contrast, neither ASIC2 nor ASIC3 knockout affect the bilayer model of mechanotransduction in the membrane deformation approach, which suggests that ASICs are not involved in mechanotransduction in the bilayer model (Drew et al., 2004) (Figure 3a).

### 3.2 Involvement of ASICs in the tether model of mechanotransduction

Compared with the bilayer model, methods to probe the tether model of mechanotransduction are more challenging, because the force needs to be specifically delivered via tethering proteins without interfering with membrane tension. One of the ways to probe the tether model of mechanotransduction is to culture neurons on an extracellular matrix‐coated elastomeric micropillar array: the focal force can be delivered by bending the pillars at up to 10‐nm scales (Poole et al., 2014). However, this elastomeric micropillar array is not easy to build and the focal force could be delivered via tethering elements and mixed with membrane tension. In 2009, Chen and colleagues developed a substrate deformation‐driven neurite stretch (SDNS) method to probe the tether model of mechanotransduction (Lin et al., 2009). In the SDNS method, neurite‐bearing neurons are cultured on an extracellular matrix‐coated elastomeric substrate (e.g., polydimethylsiloxane). Then the neurite can be stretched via substrate deformation, with an indentation pipette imposing a displacement on the substrate adjacent to (∼10–15 µm) the neurite. The indentation is controlled by a micromanipulator and the displacement can be applied with a 10‐µm step until an electrical response occurs or until a maximal displacement of 120 µm is reached (Cheng et al., 2010). In the same setting, the indentation pipette can also be placed on the neurite to induce the bilayer model of mechanotransduction. In DRG proprioceptors, SDNS could induce a mechanically activated current that was inhibited by a selective ASIC3 antagonist, APETx2, or significantly reduced in ASIC3 knockout (Lin et al., 2016) (Figure 3b). In contrast, ASIC3 knockout had no effect on the bilayer model of mechanotransduction induced by direct pipette indentation on the neurite, which suggests a modality‐specific activation of ASIC3 via the tether model of mechanotransduction in proprioceptors.

Recently, a novel mechanical stimulation using micropipette‐guided ultrasound was developed to activate ion channels involved in the tether model of mechanotransduction (Chu et al., 2021; Lim et al., 2021) (Figure 3c). The glass micropipette adapted with an ultrasound transducer is placed in the culture medium and used to apply ultrasound force to the cultured cells. A live‐cell calcium imaging assay is used to record the cellular responses to ultrasound stimuli. Ultrasound is a type of mechanical wave, so it can deliver a compression force on cells when the duty cycle is set at 100%. When the duty cycles are adjusted to 10∼30%, the micropipette‐guided ultrasound can generate two types of force with opposite directions, a pulling force from the acoustic stream and a compression force from the ultrasound wave (Lim et al., 2021). Accordingly, the micropipette‐guided ultrasound can activate an ASIC1a‐dependent tether model mechanotransduction in cultured cortical neurons or cells with heterologous expression of ASIC1a. This effect can be inhibited by selective ASIC1 antagonists (e.g., PcTx1), cytoskeletal modifiers (cytochalasin, nocodazole), or ASIC1a knockout, but not by inhibitors of Piezo ion channels (e.g., GsMTx‐4) (Lim et al., 2021). To summarize, the force modality to activate ion channels involved in the tether model of mechanotransduction differs greatly from that involved in the bilayer model of mechanotransduction (Figure 3).

## 4 UPDATING THE ROLES OF ASICS IN PROPRIOCEPTION

It is well known that the muscle spindle (MS) and Golgi tendon organ (GTO) play important roles in the sensation of active muscle contraction, passive stretch force and joint movement in the body (Proske & Gandevia, 2012; Zampieri & de Nooij, 2021). The proprioception sensation is further formed after integrating static and dynamic movement in the peripheral region and central conduction. Some mechanosensing ion channels, including ASICs, Piezo, the TRP family, transmembrane channel‐like protein and K2P, may play functional roles in proprioception (Table 2) (Lallemend & Ernfors, 2012). In DRG, parvalbumin‐positive (Pv^+^) neurons are believed to be proprioceptors. Previous studies have shown that ASIC1, ASIC1b, ASIC2a, ASIC2b and ASIC3 are expressed in Pv^+^ proprioceptive DRG neurons (Chiu et al., 2014; Lin et al., 2016). Advanced single‐cell RNA sequencing (scRNA‐seq) approaches have further provided detailed molecular elements of mechanosensing ion channels in Pv^+^ DRG proprioceptors (Table 2) (Chiu et al., 2014; Oliver et al., 2021; Usoskin et al., 2015; Wu et al., 2021).

**TABLE 2:** Expression of mechanosensing ion channels in proprioceptive parvalbumin‐positive neuron (Pv^+^) neurons in studies.

	Subtype of proprioceptor			
	(*n* = 242)	(*n* = 1109)	(*n* = 622)	Pv^+^ cluster (*n* = 92)
ASIC1	C1–5	Ia1–3, Ib, II1–4 (high)	NT4, 5 (high)	Low
ASIC2	C1–5	Ia1–3, Ib, II1–4 (high)	NT4, 5 (few)	High
ASIC3	C5 (high), C1–4 (few)	Ib, II4 (high), II1–3, Ia1–3 (low)	NT4 (medium)	High
TRPA1				Low
TRPC3	C1–5	Ia1–3, Ib, II1–3 (high)	NT5 (few)	Medium
TRPC5	C1 (few)	Ia3 (few)		Low
TRPC6		Ia1–3, II1, 2, 4 (few)		Low
TRPM7	C1–3, 5 (high), C4 (low)	Ia1–3, Ib, II1–4 (high)	NT4, 5 (medium)	Medium
TRPV1	C1, 4 (few)	Ia2, Ib, II1–3 (few)	NT4 (few)	Few
TRPV2	C1–5	Ia1, 2 (medium), Ia3 (few), Ib, II1–4 (high)	NT4 (medium), NT5 (few)	High
TRPV4	C3 (few)	Ia1–3, Ib, II2–4 (few)		Few
TMC1	C1, 5 (low)	Ia1, 3 Ib, II3 (low), Ia2, II1, 2, 4 (few)	NT5 (few)	Few
TMC2		Ia1, 2, Ib (few)		
TMHS	C1–5			
TMIE	C1, 2, 4, 5 (few)	Ia3 (few), Ia2, Ib (low), II1, 2, 4 (medium)		
TMEM120a	C1–5	Ia1–3, Ib, II1–4 (high)	NT4, 5 (medium)	Medium
TMEM150c	C1–5	Ia1–3, Ib, II1–4 (high)	NT4, 5 (low)	High
TREK1	C1, 3, 4, 5 (few)	Ia2, II2, 3, 4 (low), Ib (few), II1 (medium)		Few
TREK2	C1, 3, 5 (low), C2, 4 (few)	Ia1, 3, II1, 2, 3 (high), Ia2 (medium)	NT5 (few)	Medium
TRAAK	C3–5 (few)	Ia1 (medium), Ia2–3, Ib, II1–4 (high)	NT4 (few)	Medium
TWIK1	C1–C5	High (Ia1–3), Medium (Ib, II1–3)	NT4, 5 (high)	High
KCNMA1	C1–5	Ia1–3, Ib, II1–4 (high)	NT4 (low), NT5 (medium)	High
PKD1	C1–2 (few)	Ia1–3, Ib, II1–4 (high)	NT4 (low), NT5 (medium)	Low
PKD2	C1–5 (few)	Ia1–3, Ib, II1–4 (medium)	NT4 (few), NT5 (low)	Low
PIEZO1		Ia1–3, Ib, II2–3 (few)	NT4 (few)	Low
PIEZO2	C1–5	Ia1–3, Ib, II1–4 (high)	NT4, 5 (high)	High
Reference	Oliver et al. (2021)	Wu et al. (2021)	Usoskin et al. (2015)	Chiu et al. (2014)

*Note*: C1: type Ia afferent; C2–4: type II afferent; C5: type Ib afferent. Ia1∼3: three subtypes of type Ia afferents; II1∼4: four subtypes of type II afferents. High, medium, low, few: transcriptome level or cell number in Pv^+^ populations. According to the database of transcriptome studies focused on DRG neurons or specific proprioceptive neurons, the major channels or proteins involved in mechanotransduction in this table are highly expressed. However, the expression level may differ in subgroups of proprioceptive neurons receiving signals from other anatomical locations (type Ia, Ib and II fibres corresponding to intrafusal bag fibres, tendons of extrafusal muscles and intrafusal chain fibres). In comparing the data, ASIC2, ASIC3 and Piezo 2 are generally and highly expressed in all these four studies, which suggests that these three channels are the best candidates for proprioceptors.

### 4.1 ASIC1 and ASIC2

In 2010, Simon et al. discovered ENaC and ASIC expression and its role as mechanosensory ion channels in DRG proprioceptors (Simon et al., 2010). The authors found reduced stretch‐evoked afferent discharge, which was dose‐dependent on amiloride. Because amiloride had a broad inhibition effect on both Deg/ENaC and the ASIC family, the authors further investigated the expression of ENaC α, β and γ subunits and ASIC subtypes in MS. Both ASIC2 and ENaC co‐localized with synaptophysin, a specific marker of spindle primary afferent endings (Simon et al., 2010). Wu et al. used scRNA‐seq to reveal molecular signatures of MS and GTO. The authors found that ASIC1 and ASIC2 were universally expressed in all subgroups of Ia, Ib and II afferent fibres (Wu et al., 2021). Another scRNA‐seq study of proprioceptors showed that ASIC1 and ASIC2 were close to the afferent endings of both MS and GTO (Oliver et al., 2021). A recent study showed that ASIC2‐knockout mice exhibited skeletal malalignment and impaired proprioceptive performance in beam‐walking tasks and gait coordination, whereas the morphology of MS and GTO, locomotion abilities and treadmill walking distance were similar to that for wild‐type mice (Bornstein et al., 2023). However, the role of ASIC1 in proprioception remains to be determined.

### 4.2 ASIC3

**FIGURE 4:**
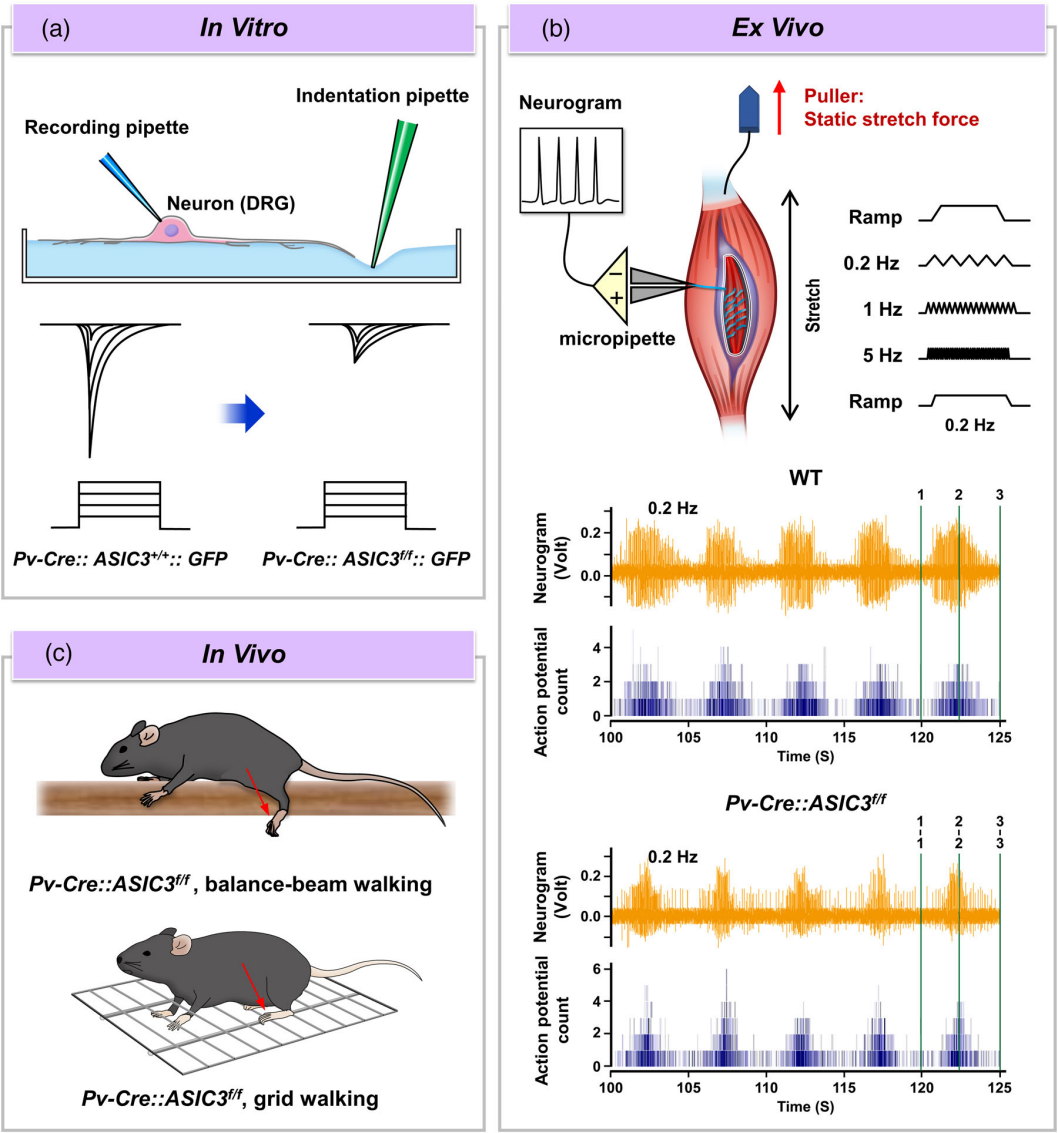
Approaches to probing the role of ASIC3 in proprioception. (a) Indentation of the substrate near the neurite, not the soma, can trigger an inward current of the *Pv‐Cre::ASIC3^+/+^::GFP* neuron, but this current is reduced in the ASIC3 conditional knockout neuron (*Pv‐Cre::ASIC3^f/f^::GFP*). (b) An *ex vivo* recording of muscle stretch‐evoked dynamic firing is used to probe the effect of ASIC3 knockout on muscle spindle activity. The soleus muscle is fixed with the lower tendon and pulled upward at the upper tendon by an electromagnetic puller to static stretch force (red arrow). Pre‐ and post‐stretch ramp stimulation are applied in this protocol. A micropipette recording neural firing activity is placed at the nerve (light blue). The potential from the neurogram and a number of action potentials (counted for potential over the background noise) are plotted as orange and dark blue, respectively. The green vertical lines, denoted 1, 2 and 3, represent pre‐stretch, holding and post‐stretch phases. The ‘On’ phase is between 1 and 1, and the ‘Off’ phase is between 2 and 3. The ACIS3 conditional knockout group shows reduced Off response with 0.2 and 1 Hz stimulation and greater On response with 1 and 5 Hz stimulation. Thus, ASIC3 deletion may enhance stretch‐evoked dynamic firing, but the firing is suppressed in the released phase. (c) Proprioception‐associated behaviours of balance‐beam and grid walking can be tested for the effect of ASIC3 knockout. The foot fault numbers of hind paws are significantly increased in ASIC3 conditional knockout mice in the behaviour tests (both balance‐beam walking and grid‐walking tasks in the dark), thus indicating local motion defects. WT, wild type.

To answer the question of the contribution of ASIC3 in proprioceptive function, Lin et al. used genetic labeling (*Pv‐Cre::GFP*) and knockout approaches (*Pv‐Cre::ASIC3^f/f^*) to dissect the roles of ASIC3 in mechanotransduction of DRG proprioceptors in vitro, *ex vivo* and in vivo (Lin et al., 2016). Whole‐cell patch clamp recordings showed that all Pv^+^ DRG neurons expressed the SDNS‐induced mechanosensitive currents, which were significantly decreased in the ASIC3‐knockout condition (Figure 4a). In an *ex vivo* setting of muscle spindle recording, ASIC3 knockout conferred abnormal dynamic responses of muscle spindles (Figure 4b). In behavioural assays, proprioceptor‐selective ASIC3‐knockout mice exhibited increased foot fault errors in balance‐beam and grid walking tests in the dark (Figure 4c), which implies impaired proprioception deficits (Lin et al., 2016). Notably, the proprioceptive deficit behaviours were not found in nociceptor‐selective ASIC3 knockout mice (*Nav1.8‐Cre::ASIC3^f/f^*). The study provided evidence that loss of the ASIC3‐mediated tether model of mechanotransduction could impair proprioception function *ex vivo* and in vivo (Figure 4).

An interesting finding from Oliver et al. using the transcriptome in Pv^+^ RunX3^+^ proprioceptive neurons from cervical to lumbar DRG in mice revealed ASIC3 predominantly expressed in type Ib afferents, but ASIC1 and ASIC2 were widely expressed in all subgroups of proprioceptor afferents (Oliver et al., 2021). Whether the result reflects the different ratios of ASIC homotrimer and heterotrimer even in the subpopulation of proprioceptive neurons needs to be further discussed and investigated. Subtype‐specific knockouts of ASIC1a, ASIC1b, ASIC2a and ASIc2b in proprioceptors are needed in future studies.

### 4.3 Other mechanosensing ion channels and associated proteins involved in proprioception

Increasing evidence indicates that other ion channels such as Piezo2, and TRP channels (Bewick & Banks, 2015) also sense and convert mechanical stimulation to electric signals in MS, GTO, and many cutaneous sensory corpuscles. Piezo2 is a non‐selective cation channel that is responsive to mechanical stimulation parallel to the membrane in parvalbumin‐expressing sensory neurons. A marked reduction in stretch inducing firing in the muscle–nerve recording from Piezo‐deficient mice indicated that Piezo2 is a principal mechanosensitive ion channel specific for proprioception (Woo et al., 2015). Compound mutations in *PIEZO2* in humans present as loss of discriminative touch perception and mechanical stimulation and severe proprioceptive impairment resulting in ataxia and dysmetria (Chesler et al., 2016).

In summary, ASIC2, ASIC3 and Piezo2 are abundantly expressed in Pv^+^ proprioceptive neurons and are likely mechanosensitive ion channels for proprioceptive signals. However, although ASIC2/3 and Piezo are gated via different force modalities, how different types of mechanically sensitive ion channels can integrate signals in the same proprioceptive nerve terminals remains to be determined.

### 4.4 Issues of controversy and unsolved questions

So far, genetic approaches of ASIC1a, ASIC2 and ASIC3 knockout have provided substantial evidence to support roles of different ASIC subtypes in neurosensory mechanotransduction, despite discrepant results, possibly due to different mechanical modalities applied in previous studies (Lin et al., 2015; Roza et al., 2004). For instance, when the membrane deformation approach was applied, ASIC2/ASIC3 double‐knockout mice showed no effect on mechanically activated currents of the bilayer model of mechanotransduction in all DRG neuron subpopulations (Drew et al., 2004). Also, functional ASICs are assembled as homomeric or heteromeric trimers. Thus, a single ASIC subtype may cause altered ASIC composition specific to a cell type, which is a possible explanation for the subtle alteration of phenotypes or unexpected phenotypes observed in knockout studies of ASIC1a, ASIC2 and ASIC3 (Lin et al., 2015). Intriguingly, one study of ASIC1a, ASIC2 and ASIC3 triple knockout showed enhanced cutaneous mechanosensitivity and enhanced activity of A‐mechanonociceptors, which suggests an unexplored role of ASIC1b in the tether model of mechanotransduction (Kang et al., 2012). Unfortunately, the proprioceptor functions were never tested in the triple knockouts. Still, we lack data on the effects of ASIC1a, ASIC1b, ASIC2a and ASIC2b knockout on neurosensory mechanotransduction of proprioceptors. To further understand the roles of ASICs in proprioception subtypes, neuron subtype‐specific knockouts of ASIC1a, ASIC1b, ASIC2a and ASIC2b are needed.

A recent study of prolotherapy may indirectly support the hypothesis of ASIC1a participating in neurosensory mechanotransduction. Injection of hypertonic 5% dextrose in muscles as a prolotherapy could reduce mechanical hyperalgesia in a mouse model of fibromyalgia induced by intramuscular acidosis (Han et al., 2022). The 5% dextrose‐mediated antinociceptive effect was volume‐dependent but osmolarity‐independent, which suggests membrane tension is not the determining factor. A mathematical model predicts that the volume‐dependent effect of dextrose can generate ∼200 kPa stress to stretch local nerves in muscle, whose force range is sufficient for ASIC activation but far lower than the thresholds of other mechanically activated ion channels of the bilayer model of mechanotransduction (Chu et al., 2021). Furthermore, the dextrose prolotherapy could be blocked by ASIC1a knockout or use of the selective ASIC1a antagonist PcTx1 as well as blockade of substance P signalling (by knockout or antagonist), but not by ASIC3 knockout or use of the ASIC3 antagonist APETx2.

Together, evidence of genetic exploration has supported a role for different ASIC subtypes in the tether model of mechanotransduction. Further studies should address how homomeric or heteromeric ASICs work in different proprioceptor subtypes and how ASICs are gated via the tethering proteins as well as how ASICs and other mechanically activated ion channels (e.g., Piezo 2) integrate mechanosensing signals in proprioceptive nerve terminals.

## 5 CLINICAL IMPLICATIONS AND FUTURE DIRECTIONS

Some questions related to the mechanism of proprioceptive defects correlated with diseases have not been resolved. Whether these defects are potential targets of ASICs or other mechanoreceptors may be a research interest in the future. For example, patients with diabetes mellitus may have large‐fibre neuropathy presenting as sensory ataxia and gait disturbance in addition to painful neuropathy (Brown et al., 2015; Vinik, 2016). The roles of ASICs contributing to diabetic neuropathy have not been established. Animal studies of streptozotocin‐induced and *db*/*db* diabetic mice revealed little pathological change except for a reduced percentage of large‐diameter neurons in DRG (Kishi et al., 2002). An immunohistochemical study of muscle spindles showed substantial variability in axonal width and inter‐rotational distance of Ia fibres in muscle spindles in both type 1 and type 2 diabetic mice (Table 3) (Muller et al., 2008). These induced diabetic mice had impaired gait balance presenting as increased foot fault in performing the beam‐walk task but not grid walking (Muller et al., 2008). Rahman et al. found hypersensitivity to pain, slowing of sensory nerve conduction velocity and upregulation of *Asic3* and *Trpv1* in DRG neurons with increased mRNA levels in streptozotocin‐induced diabetic mice, which is related to activation of pyruvate dehydrogenase kinase and accumulation of intracellular lactic acid (Rahman et al., 2016). Beyond pain modulation, whether the proprioceptive DRG function regulated by ASIC3 is affected in diabetes needs further investigation.

**TABLE 3:** Diseases associated with proprioceptive dysfunction.

Diseases	Clinical presentation	Electrodiagnosis	Pathogenic location	Animal model	Impact
Diabetic neuropathy/diabetic pseudotabes	Hypoesthesia; sensory ataxia ± neuropathic pain ± autonomical neuropathy	NCS: reduced SNAP amplitude/sensory NCV; nerve excitability test; microneurography?	Large myelinated fibres (Aα, Aβ), DRG, muscle spindle	STZ‐induced diabetic mice: axonal loss, large DRG neurons reduced (Kishi et al., 2002), afferent in muscle spindle derangement (Muller et al., 2008; Muramatsu et al., 2017; van Deursen et al., 1998)	Falling, trauma, fracture, Charcot's arthropathy
Vitamin B6 (pyridoxine) intoxication	Sensory ataxia, pseudoathetosis, altered sensation without motor weakness	NCS: severely reduced SNAP amplitude or absence of SNAP; SSEP: impairs central and peripheral conduction	Large myelinated fibres, posterior column of the spinal cord	Rat: necrosis of DRG, especially large neurons, degeneration of large myelinated axons (Perry et al., 2004); dog: degeneration of DRG, dorsal roots and peripheral nerves (Chung et al., 2008)	Falling, gait disturbance because of reversible neurological deficit
Vitamin B12 deficiency/SCD	Sensory ataxia without motor weakness	SSEP: impaired central and peripheral conduction; NCS: slowing of NCV	Posterior column of the spinal cord, large myelinated fibre	Transcobalamin receptor knockout mouse (Cd320^−/−^) (Arora et al., 2019)	Unbalanced gait
CIPN	Hyperalgesia; sensory ataxia	NCS: reduced SNAP amplitude; nerve excitability test	Small unmyelinated fibres, large myelinated fibres	Taxol injected rat: dorsal root hypomyelination, ↓ H reflex amplitude, impaired balance‐beam walking (Cliffer et al., 1998); oxaliplatin functionally impaired sustained spindle afferent firing during static muscle stretch without axonal injury (Bullinger et al., 2011)	Unbalanced gait, cancer treatment efficiency because of the side effects of chemotherapies
Sensory CIDP, anti‐MAG associated CIDP	Relapse‐remitting or progressive motor weakness with sensory ataxia, limbs tremor	NCS: slowing of sensory and motor NCV, even conduction block; nerve excitability test	Large myelinated fibres	Mice or rat EAN model (Soliven, 2014) but not specific for sensory type CIDP	Unbalanced gait
Autoimmune/paraneoplastic sensory neuronopathy	Sensory ataxia, loss of touch and vibratory perception, motor dysfunction	NCS: reduced amplitude of SNAP, relative sparing sensory latencies and NCV	DRG degeneration or posterior column of the spinal cord	Inflammation and loss of ganglion cells in the dorsal root ganglia (Kusunoki et al., 1999; Ohsawa et al., 1993)	Unbalanced gait
CMT (type 1A and 2E)	Distal limb weakness, sensory ataxia, ± foot deformity	NCS: slowing of NCV, reduced SNAP amplitude	Large myelinated fibres	*Swl* mutant mice: early‐onset muscle spindle deficiency (Chen et al., 2007); hNF‐L^E396K^ transgenic mice (Villalon et al., 2017)	Posture instability, foot deformity/Charcot joint
Neurosyphilis (Tabes dorsalis)	Severe sensory ataxia, extensor muscle contraction, pupillary dysfunction	SSEP: impaired central and peripheral conduction	Dorsal column of the spinal cord, large myelinated fibres		Joint degeneration, fracture, loss of coordination

*Note*: This table summarizes the neurological diseases along with proprioceptive dysfunction and sensory ataxia. According to electrodiagnostic and animal studies, most diseases involve large myelinating fibres in the peripheral nerves, DRG neurons and posterior column of the spinal cord. The roles of ASICs, other channels or proteins in these disorders remain unclear, and further investigations focusing on ASICs and other proteins are essential. Abbreviations: ↓, decreased or impaired; anti‐MAG, anti‐myelin‐associated glycoprotein; CIDP, chronic inflammatory demyelinating polyradiculoneuropathy; CIPN, chemotherapy‐induced peripheral neuropathy; CMT, Charcot–Marie–Tooth disease; EAN, experimental allergic/autoimmune neuritis; hNF‐L, human neurofilament light chain; NCS, nerve conduction study; NCV, nerve conduction velocity; SCD, subacute combined degeneration; SNAP, sensory nerve action potential; SSEP, somatosensory evoked potential; STZ, streptozotocin.

Furthermore, sensory neuronopathy involving DRG may be another target of the translational study model for proprioception and mechanoception. Sensory neuronopathy presenting ataxia, gait disturbance, hyporeflexia and cutaneous hypoesthesia without weakness may result from nutritional deficiency (vitamin B12, folic acid; McCombe & McLeod, 1984), toxin exposure (chemotherapy agents, Bullinger et al., 2011; pyridoxine overdose, Schaumburg et al., 1983; nitrous oxide, Swart et al., 2021), a paraneoplastic effect or autoimmune diseases (Sjögren's syndrome, chronic inflammatory demyelinating polyradiculoneuropathy; Amato & Ropper, 2020; Mathis et al., 2021). Table 3 lists diseases involving proprioceptive dysfunction. These diseases affecting large fibre and proprioceptive function could be used to explore the function of proprioceptive DRG neurons and the expression levels of key mechanosensing ion channels. Although the pathology of these diseases has been established, the molecular mechanism involving the ion channels for proprioception and mechanotransduction should be clarified clearly.

## 6 CONCLUSIONS

ASICs are specific ion channels activated by protons, non‐proton ligands and mechanical stimulation. They are widely distributed in various tissues, including the nervous system, cutaneous sensory corpuscles and other non‐neuronal cells. The use of genetic manipulation such as ASIC knockout, advanced electrophysiological recording studies, ultrasound techniques and the tether model of mechanotransduction would give a better understanding of how ASICs contribute to mechanotransduction, including proprioception. Despite lack of information on the role of ASICs in proprioceptive disorders, investigating the relationship between ASICs and peripheral nervous diseases may be a new direction in the future.

## AUTHOR CONTRIBUTIONS

Yi‐Chen Lin, Cheng‐Han Lee, Jia‐Ying Sung, and Chih‐Cheng Chen designed the concept of this article and drafted the manuscript. Yi‐Chen Lin and Chih‐Cheng Chen contributed to the revised edition. All authors have read and approved the final version of this manuscript and agree to be accountable for all aspects of the work in ensuring that questions related to the accuracy or integrity of any part of the work are appropriately investigated and resolved. All persons designated as authors qualify for authorship, and all those who qualify for authorship are listed.

## CONFLICT OF INTEREST

None declared.
